# Ecdysteroids Sensitize MDR and Non-MDR Cancer Cell Lines to Doxorubicin, Paclitaxel, and Vincristine but Tend to Protect Them from Cisplatin

**DOI:** 10.1155/2015/895360

**Published:** 2015-05-17

**Authors:** Ana Martins, Péter Sipos, Katalin Dér, József Csábi, Walter Miklos, Walter Berger, Attila Zalatnai, Leonard Amaral, Joseph Molnár, Piroska Szabó-Révész, Attila Hunyadi

**Affiliations:** ^1^Department of Medical Microbiology and Immunobiology, Faculty of Medicine, University of Szeged, Dóm tér 10, Szeged 6720, Hungary; ^2^Unidade de Parasitologia e Microbiologia Médica, Instituto de Higiene e Medicina Tropical, Universidade Nova de Lisboa, Rua da Junqueira 100, 1349-008 Lisbon, Portugal; ^3^Department of Pharmaceutical Technology, Faculty of Pharmacy, University of Szeged, Eötvös Ucta 6, Szeged 6720, Hungary; ^4^Institute of Pharmacognosy, Faculty of Pharmacy, University of Szeged, Eötvös Ucta 6, Szeged 6720, Hungary; ^5^Department of Medicine I, Institute of Cancer Research and Comprehensive Cancer Center, Medical University of Vienna, Borschkegasse 8A, 1090 Vienna, Austria; ^6^Institute of Inorganic Chemistry and Research Platform “Translational Cancer Therapy Research”, University of Vienna, Waehringer Strasse 42, 1090 Vienna, Austria; ^7^First Department of Pathology and Experimental Cancer Research, Semmelweis University, Üllői út 26, Budapest 1085, Hungary; ^8^Centro de Malária e Outras Doenças Tropicais (CMDT), Instituto de Higiene e Medicina Tropical, Universidade Nova de Lisboa, Rua da Junqueira 100, 1349-008 Lisbon, Portugal

## Abstract

Ecdysteroids, analogs of the insect molting hormone, are known for their various mild, nonhormonal bioactivities in mammals. Previously, we reported that less-polar ecdysteroids can modulate the doxorubicin resistance of a multidrug resistant (MDR) mouse lymphoma cell line expressing the human ABCB1 transporter. Here, we describe the ability of 20-hydroxyecdysone (**1**) and its mono- (**2**) and diacetonide (**3**) derivatives to sensitize various MDR and non-MDR cancer cell lines towards doxorubicin, paclitaxel, vincristine, or cisplatin. Drug IC_50_ values with or without ecdysteroid were determined by MTT assay. Compound **3** significantly sensitized all cell lines to each chemotherapeutic except for cisplatin, whose activity was decreased. In order to overcome solubility and stability issues for the future *in vivo* administration of compound **3**, liposomal formulations were developed. By means of their combination index values obtained via checkerboard microplate method, a formulation showed superior activity to that of compound **3** alone. Because ecdysteroids act also on non-ABCB1 expressing (sensitive) cell lines, our results demonstrate that they do not or not exclusively exert their adjuvant anticancer activity as ABCB1 inhibitors, but other mechanisms must be involved, and they opened the way towards their *in vivo* bioactivity testing against various cancer xenografts.

## 1. Introduction

Ecdysteroids represent a particularly interesting group of natural compounds from several aspects, with functions in all kingdoms of nature: in insects, they play a crucial hormonal role controlling molting and development [1]; in plants, they appear to serve as part of the chemical defense against nonadapted herbivores [2]; and, although with a less-studied and unclear role, they are also present in fungi [3]. These compounds show fundamental differences to the mammalian steroid hormones, which make them unable to interact with their hormonal system [4]. Despite this, a number of rather beneficial metabolic effects have been attributed to them in mammals including humans: a mild anabolic activity of ecdysteroids undoubtedly exists [4], and these compounds can also influence both glucose and lipid homeostasis [5], altogether resulting in a so-called adaptogenic or “antistress” effect.

The role of ecdysteroids in cancer is yet to be understood. In accordance with their “general strengthening” effect on mammals,* in vitro* antiapoptotic effect of muristerone A was observed in RKO human colon carcinoma cells [6]. As a result of a thorough study on many natural and semisynthetic ecdysteroids, we have recently reported that certain derivatives can significantly decrease the doxorubicin resistance of a multidrug resistant (MDR) mouse lymphoma cell line (L5178_MDR_) that has been transfected with the pHa MDR1/A retrovirus to express the human ABCB1 or P-glycoprotein, an ATP-binding cassette (ABC) transporter [7]. Mild to strong synergism with doxorubicin was found for the less-polar derivatives, while classical, polar ecdysteroids, such as, for example, 20-hydroxyecdysone (20E;** 1**), could act in a weak antagonism or indifferent way with this chemotherapeutic agent [7]. Our following structure-activity relationship studies revealed that the introduction of apolar groups at the 20,22 and particularly at the 2,3 position is of key importance in order to have a sensitizing effect on doxorubicin in the aforementioned cell line [7–9]. Interestingly, although several of the less-polar ecdysteroids could inhibit the function of ABCB1, this inhibition was moderate or negligible and only a very marginal correlation to the strength of synergism with doxorubicin could be found [7].

**Figure 1:**
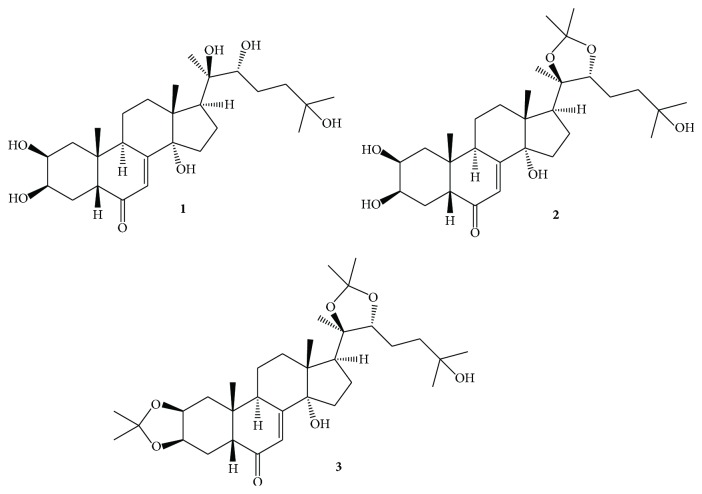
The structures of compounds** 1**,** 2**, and** 3**.

Two derivatives of 20E (**1**), 20-hydroxyecdysone 20,22-acetonide (**2**), and 20-hydroxyecdysone 2,3;20,22-diacetonide (**3**) are of particular interest in our studies; structures of these three compounds are shown in Figure 1.

Compounds** 2** and** 3** can also be found in the nature but their semisynthetic preparation from the abundant 20E (**1**) is extremely simple and economic; moreover, compound** 3** was among the most active ecdysteroids in our previous studies [7–9]. In fact, compounds** 1**–**3** represent good examples for the different levels of activity of ecdysteroids in L5178_MDR_ cells from the weak antagonism to the strong synergism when coadministered with doxorubicin, which makes these three compounds an ideal choice to further study the effects of ecdysteroids in cancer and also the mechanism by which they exert their activity. On the other hand, the well-known acid sensitivity of acetonide groups (resulting in a quick decomposition of compound** 3** to compound** 2** at gastric pH [7]) and solubility problems, mainly of compound** 3**, could be an impediment to further* in vivo* studies. This fact made further formulations necessary prior to performing animal studies.

Nanosized drug delivery systems, such as liposomes, are potential carriers for the encapsulation of bioactive agents, both hydrophilic and hydrophobic molecules, peptides, and so forth. Synthetic and natural phospholipids and cholesterol derivatives are important components of the biocompatible, less immunogenic, and nontoxic liposomes [10, 11]. Controlled or targeted drug release and reduction of the number and strength of side effects are the main advantages.

In the present paper, we report the investigation of compounds** 1**–**3** in combination with various chemotherapeutic agents against a panel of different drug-sensitive and drug-resistant cancer cell lines, as well as the development of a liposomal formulation of compound** 3** in order to allow future* in vivo* studies.

## 2. Materials and Methods

### 2.1. Chemicals and Reagents

20E (90% purity, originating from the roots of* Cyanotis arachnoidea*) was purchased from Shaanxi KingSci Biotechnology Co. Ltd. (Shanghai, China) and further purified by crystallization to possess 97.8% purity; this served as the starting material for the semisynthesis of compounds** 2** and** 3** as published before [8]. Phosphatidylcholines as L-alpha phosphatidylcholine and lecithin and 1,2-distearoyl-*sn*-glycero-3-phosphoethanolamine-N-[methoxy(polyethylene glycol)-3000] (ammonium salt) as PEG-3000 PE were purchased from Avanti Polar Lipids Inc. (US). Cholesterol, polyoxyethylene-24-cholesterly ether, beta-D-glucopyranoside, anhydrous ethanol, sodium chloride, and sodium phosphate buffer salt (Na_2_HPO_4_·2H_2_O) were purchased from VWR Int. Ltd. (Austria). PES syringes and membrane syringe filters with pore sizes of 100, 220, and 900 nm and a diameter of 25 mm were purchased from Phenomenex Inc. (Gen-Lab Ltd., Hungary).

### 2.2. Preparation of Liposomes

Liposomes were prepared by the hydration of thin dry lipid film method. The compositions used were selected according to a preliminary preformulation study (in preparation). Briefly, L-alpha phosphatidylcholine (L-PC), cholesterol, lecithin, polyoxyethylene-24-cholesterly ether (C-24), and beta-D-glucopyranoside or PEG-3000 PE were solubilized together with compound** 3** in anhydrous ethanol at 65°C; compositions of formulae LIP-1, LIP-2, and LIP-3 are shown in Table 1. Polyoxyethylene-24-cholesterly ether, beta-D-glucopyranoside (b-DG), and cholesterol were used to stabilize the liposome bilayer. The PEG-3000 PE was used as material for PEGylation of the liposomes. The solvent was later removed by evaporation on a rotary vacuum evaporator (Büchi Rotavapor R-210 System, Büchi Labortechnik, Switzerland) at *p* = 150 mbar. The lipid film layer was rehydrated in filtered (100 nm) phosphate buffer saline solution (pH 7.4) having the osmolality of 279 mOsm/L. The liposome dispersion was kept in a refrigerator at 4°C for 2 h prior to the droplet size reduction. The process was achieved by sonicating the liposome dispersion for 15 min at 100% amplitude (Elma TH075EL Ultrasonic bath, ELMA GmbH & Co. KG, Germany). Liposomes of uniform size were prepared by filtration (membrane filters, PES) once through 900 and 220 nm pore size filters. Sorbic acid (0.05% w/v) was added to the buffer solution in order to ensure microbiological stability.

### 2.3. Determination of the Average Hydrodynamic Size and Surface Electric Charge of Liposome Dispersion

The liposome dispersion was characterized for average droplet size, polydispersity index (PDI, representing size distribution), and surface electric charge by DLS (dynamic laser scattering) method with a Zetasizer Nano ZS (Malvern Instruments Ltd., UK). The optical parameters (i.e., refractive index of the dispersion and the buffer solution) and the conductivity were measured before the DLS measurement. The liposomes were stored at 25°C for 1 h prior to analysis and then diluted with ultrapure water. Liposomes were measured after 1 week storage time, which is enough to observe the possible phase separation (i.e., the conflux of liposome droplets) that could be caused by any undesirable alteration of the preparation process (*n* = 3).

### 2.4. Quantitative Analysis of Compound **3** within Liposomes

0.5 mL of LIP-1, LIP-2, or LIP-3 was measured into a 5.0 mL volumetric flask, diluted to 1.5 mL with HPLC-gradient water and adjusted to 5.0 mL with HPLC grade methanol. HPLC analysis was performed on a system of two Jasco PU-2080 pumps connected to a Jasco MD-2010 Plus photodiode-array detector, in isocratic mode by 70% aqueous methanol on a Zorbax XDB-C8 column (5 *μ*m, 4.6 × 150 mm) at a flow rate of 1.5 mL/min at *λ* = 243 nm. Each measurement was performed in triplicates, as well as the calibration that was performed by injecting 0.2, 0.5, 1.0, 3.0, and 5.0 *μ*g of compound** 3**.

### 2.5. Cell Lines

Six human derived cancer cell lines were used in this study: breast cancer MCF7 cells and their subcell line obtained by adaptation to doxorubicin, MCF7_dox_ [12] cultured in EMEM media supplemented with nonessential amino acids, 1 mM Na-pyruvate, and 10% inactivated fetal bovine serum (MCF7_dox_ was cultured in the presence of 1 *μ*M of doxorubicin each third passage); prostate cancer cells PC3 and LNCaP cultured in RPMI 1640 media supplemented with 10% inactivated fetal bovine serum; in case of LNCaP medium also contained 1 mM Na-pyruvate, HEPES, and glucose; epidermal carcinoma cell line KB-3-1 and its subline KB-C-1 obtained by stepwise adaption to colchicine (generously donated by D. W. Shen, Bethesda, USA) cultured in RPMI 1640 media supplemented with 10% inactivated fetal bovine serum. All cells were cultured at 37°C and 5% CO_2_; all media contained nystatin, 2 mM L-glutamine, 100 U penicillin, and 0.1 mg streptomycin.

Two mouse lymphoma cell lines were also used: a parental (L5178) cell line, L5178 mouse T-cell lymphoma cells (ECACC catalog number 87111908, U.S. FDA, Silver Spring, MD, U.S.), and a multidrug resistant (L5178_MDR_) cell line derived from L5178 by transfection with pHa MDR1/A retrovirus [13]. Cells were cultured in McCoy's 5A media supplemented with nystatin, L-glutamine, penicillin, streptomycin, and inactivated horse serum, at 37°C and 5% CO_2_. MDR cell line was selected by culturing the infected cells with 60 *μ*g/L colchicine (Sigma).

Media, fetal bovine serum, horse serum, and antibiotics were purchased from Sigma.

### 2.6. Cytotoxicity Assays

*Cytotoxic Activity.* Cytotoxic activities were evaluated by serial-dilution method in 96-well flat-bottom microtiter plates. In case of L5178 and L5178_MDR_ cell lines, 6 × 10^3^ cells were added to each well and results were evaluated using 10% MTT after 72 h incubation at 37°C, 5% CO_2_. In case of PC3 and LNCaP cell lines, 1 × 10^4^ cells were added to each well and results were evaluated using 10% MTT after 48 h. With respect to the MCF7, MCF7_DOX_, KB-3-1, and KB-C-1 cell lines, 1 × 10^4^ cells per well were seeded overnight and serial dilutions of the compound were added the following day and incubated for 48 h or 72 h. In all cases, the precipitate formed in the MTT reaction was diluted with 10% SDS-HCl after 4 h. Plates were incubated overnight and optical density was measured at 540 and 630 nm using an ELISA reader (Multiskan EX, Lab Systems, U.S.). Fifty per cent inhibitory concentrations (IC_50_) were calculated using nonlinear regression curve fitting of log(inhibitor) versus normalized response and variable slope with a least squares (ordinary) fit of GraphPad Prism 5 software.

### 2.7. Combination Studies at Fixed Ecdysteroid Concentration

As compounds** 1**–**3** were found to exert very low cytotoxicity activity on each cell line (see below), the activity of 50 *μ*M of compound on the IC_50_ of doxorubicin (Teva), paclitaxel (Mayen) cisplatine (Teva), or vincristine was tested using the same protocol as described above to the cytotoxicity testing for the respective cell lines. In each case, statistical analysis was carried out by one-way ANOVA with Bonferroni's post hoc test, and differences were considered significant at ^*^ *P* < 0.05, ^**^ *P* < 0.01, and ^***^ *P* < 0.001. In order to prevent any possible false positive results and strengthen the relevance of our data for possible* in vivo* studies, we also set up a stricter criterion: statistically significant potentiation was considered relevant only when at least a two-time decrease in the IC_50_ of the chemotherapeutic agent was observed. Such measures were not applied in case of an antagonistic effect (see results for cisplatin).

### 2.8. Combination Studies with Liposomes Containing Compound **3**

The combined activity of doxorubicin (Teva) and formulations LIP-1, LIP-2, and LIP-3 on the L5178_MDR_ mouse lymphoma cells was determined using the checkerboard microplate method in 96-well flat-bottom microtiter plates as published before [7]. Briefly, 1 × 10^4^ cells/well were incubated with different concentrations of doxorubicin and liposomes containing compound** 3** for 48 h at 37°C under 5% CO_2_. Cell viability rate was determined through MTT staining, and the interaction was evaluated by using the CompuSyn software (CompuSyn, Inc., U.S.) for the constant liposome versus doxorubicin ratios. Combination index (CI) values are presented for 50%, 75%, and 90% of growth inhibition. In each case, the amounts of liposomes were applied so that they represent the same amount of compound** 3** within, based on the results of the quantitative determination by HPLC (see above, Section 2.4). As such, M/M ratios of compound** 3** versus doxorubicin were used to calculate the results, which was performed according to that suggested by Chou [14].

## 3. Results and Discussion

Our previous observations on the strong activity of compound** 3**, a less-polar ecdysteroid, against the doxorubicin resistance of ABCB1 expressing MDR murine lymphoma cells [7] led to the need of a thorough study on these compounds using various human cell lines and chemotherapeutics. The chosen cell lines included drug susceptible/MDR cell line pairs, such as the previously used L5178 and the ABCB1 transfected L5178_MDR_ (mouse lymphoma), MCF7 and its ABCB1 expressing subcell line MCF7_dox_ adapted to doxorubicin (mammary gland), and KB-3-1 and its ABCB1 expressing subcell line KB-C-1 adapted to colchicine (cervix). Two prostate cancer cell lines were also used in our experiments, the steroid dependent LNCaP and the nonsteroid dependent PC3. When tested alone on these cell lines, compounds** 1**,** 2**, and** 3** exerted very low cytotoxic activities with IC_50_ values of typically >100 *μ*M, except for compound** 3** on the L5178 (81.12 ± 5.5 *μ*M) and compound** 2** on the MCF7 cell line (89.67 ± 4.1 *μ*M).

**Figure 2:**
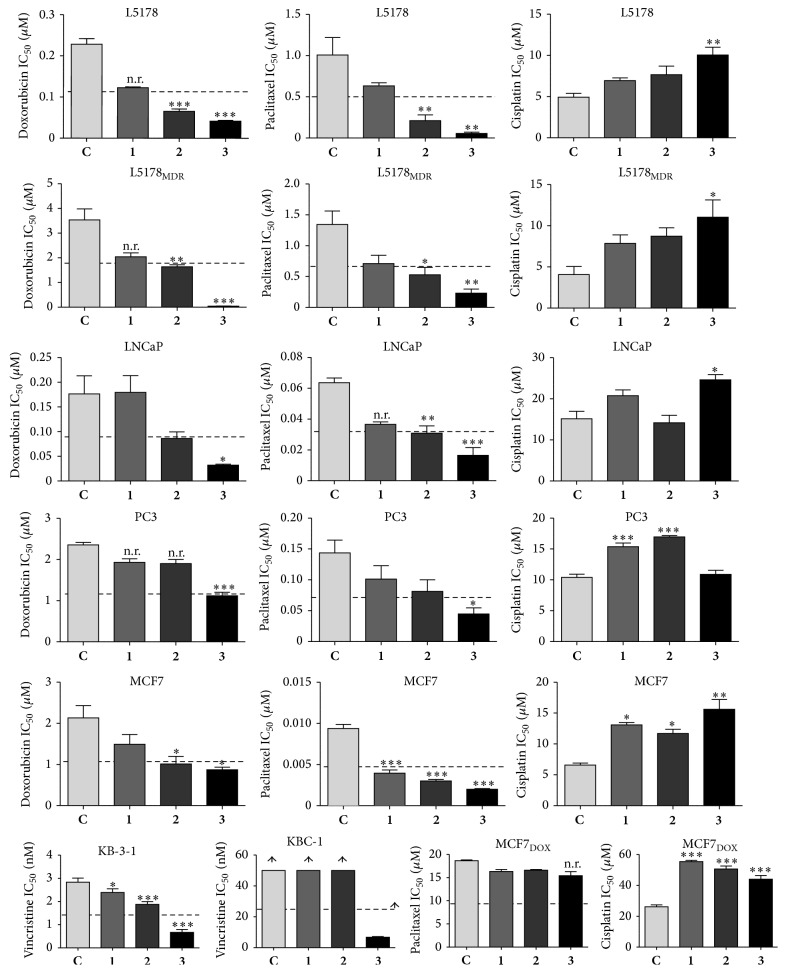
The effect of 50 *μ*M of** 1**,** 2**, or** 3** on the IC_50_ values of chemotherapeutics in various susceptible and MDR cell lines. ^*^ *P* < 0.05, ^**^ *P* < 0.01, and ^***^ *P* < 0.001 by means of one-way ANOVA followed by Bonferroni post hoc test as compared to that of the chemotherapeutic agent alone (**C**); n.r.: statistically significant, but not relevant sensitization.

The effect of 50 *μ*M of compounds** 1**–**3** was tested on the cytotoxic activity of chemotherapeutics with distinct mechanisms of action, such as doxorubicin (topoisomerase II inhibitor, ABCB1 substrate), paclitaxel (stabilizes microtubule polymer, ABCB1 substrate), vincristine (antitubulin agent on tubulin dimers, ABCB1 substrate), and cisplatin (alkylating agent, non-ABCB1 substrate); results are shown in Figure 2.

It is important to mention that even though compounds** 1**–**3** exert negligible intrinsic cytotoxic activity at the applied concentration of 50 *μ*M, this approach is still a simplification as compared to an appropriate calculation of synergism, for example, by using the checkerboard plate method [14], and, as such, it could lead to false positive results. Considering this and also taking into account our previous observations on the IC_50_ patterns in combination studies on checkerboard plates [7], we decided to accept only those results as actual sensitizing activity, where at least a two-time decrease in the IC_50_ value of the chemotherapeutic could be observed.

Compound** 3** exerted a significant sensitization effect on all cell lines when applied together with doxorubicin, vincristine, or paclitaxel. Results with doxorubicin could not be determined on the highly resistant MCF7_dox_ cell line since that chemotherapeutic agent had to be applied in such high doses that the measurements were disturbed by its own color (data not shown); paclitaxel resistance of this cell line could also not be reverted. Altogether, the results that are in agreement with those observed by us previously and strongly support our previous assumption [7] that less-polar ecdysteroids do not or not exclusively act as ABCB1 inhibitors: compound** 3** could effectively sensitize non-MDR human cell lines with no detectable (MCF7 [12, 15], KB-3-1 [16, 17]) or very low (LNCaP [18]) expression of the ABCB1 protein, as well as the drug-sensitive mouse lymphoma cell line (L5178). Moreover, the IC_50_ of doxorubicin in the L5178 cell line and its ABCB1 transfected counterpart L5178_MDR_ was decreased to practically the same values, 41.7 and 45.6 nM, respectively (no significant difference by unpaired *t*-test), in the presence of 50 *μ*M of compound** 3**, while the IC_50_ values for doxorubicin alone on the L5178 and the L5178_MDR_ cell lines were 228.3 and 3537 nM, respectively. Compound** 3** was also able to reduce the paclitaxel resistance of PC3 cells to the same level of those of untreated LNCaP cells (IC_50_ = 44.8 and 63.5 nM, resp. and no significant difference by unpaired *t*-test), while the treatment of LNCaP cells with compound** 3** led to an even stronger cytotoxic activity of paclitaxel (IC_50_ = 16.5 nM). Moreover, KB-3-1 cells were sensitized by compound** 3** to vincristine in a way that it reached much lower IC_50_ values than observed in KB-C-1 cells treated with the combination: the IC_50_ value was 0.67 nM (without compound** 3**: 2.84 nM) in case of KB-3-1, while it was ca. 10 times higher, 6.78 (without compound** 3**: >50 nM) in case of the MDR subline KB-C-1. A comparison of these values also reveals that resistance of KB-C-1 cells to vincristine could not completely be reverted by compound** 3**: even though a strong sensitizing activity was observed, the cytotoxicity of vincristine was still significantly weaker than in the parental KB-3-1 cell line (*P* < 0.001 by unpaired *t*-test).

The 20,22-acetonide compound (**2**) showed tendencies for an activity pattern similar to the diacetonide** 3**, but with much weaker and in several cases irrelevant activities. Interestingly and somewhat unexpectedly, compound** 1**, 20-hydroxyecdysone, was also found to show significant and relevant sensitizing activity in case of one cell line, MCF7, when coadministered with paclitaxel.

On the other hand, the tested ecdysteroids showed an obvious general tendency to decrease the activity of cisplatin in all cell lines, especially in the two breast cancer cells lines (MCF7 and MCF7_dox_) where all compounds significantly elevated its IC_50_ values. In case of the two prostate carcinoma cell lines, the compounds showed different behaviors: only compound** 3** could significantly protect the steroid dependent LNCaP cells from the effect of cisplatin, while compounds** 1** and** 2** exerted such an activity in the PC3 cells where compound** 3** did not influence the efficacy of cisplatin. Although androgen hormone dependency is a major difference between these two cell lines, it should be noted that ecdysteroids do not exert androgenic activity [4]. Relevance of our results in terms of a possible interference with actual chemotherapy with cisplatin in cancer patients will have to be clarified by further studies; considering the large number of food supplements containing high amounts of ecdysteroids (mainly compound** 1**) available on the market, the possibility of unwanted interactions cannot be excluded. On the other hand, Konovalova et al. have previously found that 10 mg/kg of compound** 1** could potentiate the activity and decrease the toxicity of cisplatin in P388 leukemia or B16 melanoma bearing mice, and the authors suggested that beneficial metabolic and immune system modulatory effects of this compound might be the reason for this phenomenon [19]. Such mechanisms could indeed overwrite an otherwise antagonistic effect observed in our experimental* in vitro* setup. Nevertheless, the strong potentiating activity of compound** 3** on the activity of doxorubicin, vincristine, and paclitaxel on various cell lines is highly promising.

As the next step towards animal experiments, the slight acid sensitivity of compound** 3** (resulting in its decomposition to compound** 2** with a half-life of* ca.* 7.30 min at gastric pH [7]), as well as solubility problems attributed to this compound needed to be solved; in order to fulfill these objectives, liposomal formulations were developed.

The average hydrodynamic size and surface electric charge (zeta potential) parameters of liposome samples of various lipid compositions are shown in Table 2. The droplet size and the PDI values are typical of SUV (small unilamellar vesicle) type liposomes, ranging from 83 to 92 nm and from 0.16 to 0.19, respectively. One size fraction of liposomes was observed in the range of 20–220 or 20–300 nm, irrespective of the lipid composition used for the preparation. A slight increase in the zeta potential from −2.16 (LIP-1) and −2.46 (LIP-2) mV to the value of −1.37 mV (LIP-3) was observed with the addition of PEG-PE lipid.

Encapsulation of compound** 3** into LIP-1, LIP-2, and LIP-3 resulted in entrapment efficiencies of 43, 8, and 50%, respectively (Table 2). LIP-1 and LIP-2 represent different compositions, containing more L-PC and lecithin (LIP-1) or more cholesterol and C-24 (POE-cholesteryl-ether) (LIP-2). Cholesterol and C-24 were used as bilayer stabilizers, but their increased concentration was not enough to achieve complete droplet stability for LIP-2. However, the compositions of LIP-2 and LIP-3 are rather similar; the difference is the addition of the PEG-3000 PE component in the latter case. The increase of the entrapment efficiency from 8% to 50% could be due to the fact that the exact average molecular weight of PEG-3000 PE material is around tenfold higher (3772.36) than those of the other components. This relatively high molecular weight could ensure better physicochemical stability and therefore a much higher entrapment of compound** 3 **[20].

**Figure 3:**
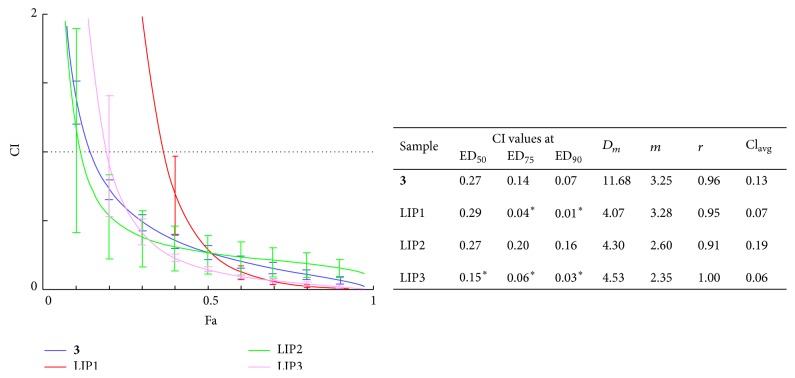
Fa-CI plots of the nonformulated compound** 3** and that enclosed within liposomes (LIP-1, LIP-2, and LIP-3) tested on the L5178_MDR_ cell line. Concentrations correspond to compound** 3**/doxorubicin ratio of 20.4 M/M in each case. Fa = fraction affected. Dashed horizontal line shows CI = 1, and CI < 1, CI = 1, and CI > 1 represent synergism, additivity, and antagonism, respectively. The error bars show 95% confidence intervals by means of serial deletion analysis. In the table, CI values are presented at 50%, 75%, and 90% of inhibition (ED_50_, ED_75_, and ED_90_, resp.); CI_avg_ = (CI_50_ + 2CI_75_ + 3CI_90_)/6; *D*_*m*_, *m*, and *r* represent antilog of the x-intercept, slope, and linear correlation coefficient of the median-effect plot, respectively. These parameters indicate the activity (IC_50_), shape of the dose-effect curve, and conformity of the data, respectively [7]; ∗: significantly stronger synergism with doxorubicin as compared to compound** 3** by means of nonoverlapping confidence intervals.

In order to investigate the activity of the liposomes containing compound** 3** in comparison with that of the nonformulated compound, interactions between LIP-1, LIP-2, and LIP-3 and doxorubicin were tested on checkerboard plates. This experimental setup was chosen because in these complex systems there were too many factors that could have influenced the results, and hence we decided not to apply the simplification presented above. Synergism/antagonism was quantified by using the CompuSyn software as in our previous work [7, 9]. Quantitative determination of compound** 3** within the formulations allowed us to apply the same doses in each case, as expressed in compound** 3** equivalents. Results of these experiments are shown in Figure 3.

Based on our results, the formulations not only keep the strong synergistic activity of compound** 3** and doxorubicin, but also, particularly in case of LIP-3, are able to show favorable activity as compared to the free compound. The slight increase in the potentiating activity might be explained by the liposomes ability to fuse with the cell membranes and deliver the enclosed compound to the cells more efficiently even* in vitro*; however, the favorable effect on the solubility of compound** 3** in the aqueous environment can also be among the reasons for this phenomenon. It is also important to note that the acidic pH of around 5 maintained within the lysosomes [21] (that compound** 3** likely faces upon the phagocytosis of the liposomes) is apparently not enough to hydrolyze the acetonide groups, which could have been a serious pitfall for these formulations if compound** 3** would decompose to the much less active compound** 2** [7].

## 4. Conclusions

We demonstrated that the 20,22-acetonide (**2**) and in particularly the 2,3;20,22-diacetonide (**3**) derivatives of 20-hydroxyecdysone (**1**) are able to exert sensitizing activity to doxorubicin, paclitaxel, and vincristine in MDR cell lines, which express the ABCB1 membrane transporter, as well as in their susceptible counterparts. Even though, in this work, no conclusions can be made about the mechanism(s) of action of these compounds, these results strongly support our previous assumptions [7] that these ecdysteroids do not or not only act as ABCB1 inhibitors when exerting their adjuvant anticancer activity.

Moreover, all ecdysteroids showed significant protective effects towards cisplatin treatment on some of the cell lines tested* in vitro*, highlighting the importance of further studies in this direction.

Compound** 3** enclosed in liposomes (LIP-3) showed stronger synergistic activity in combination with doxorubicin on L5178_MDR_ cells as compared to the case when it was applied in solution. Based on these results, LIP-3 represents a highly prospective formulation for* in vivo* studies. Such studies have most recently started within our research program; results are going to be presented in the near future.

## Figures and Tables

**Table 1:** Composition of liposome samples LIP-1, LIP-2, and LIP-3.

Components	LIP-1 (mol%)	LIP-2 (mol%)	LIP-3 (mol%)
Compound **3**	9	8	8
L-PC	44	24	25,9
Lecithin	21	15	16
C-24	7	12	12,8
b-DG	3	7	0
PEG-3000 PE	0	0	0,3
Cholesterol	16	34	37

**Table 2:** Droplet size characteristics and encapsulation efficiency of compound **3** within liposome samples LIP-1, LIP-2, and LIP-3 (*n* = 3).

Liposome samples	Average hydrodynamic size [nm]	Droplet size distribution	Polydispersity index (PDI)	Surface electric charge [mV]	Entrapment efficiency (%)
LIP-1	92.1 ± 0.5	30–300 nm (100%)	0.16	−2.16 ± 0.77	43%
LIP-2	83.2 ± 0.9	20–200 nm (100%)	0.16	−2.46 ± 0.45	8%
LIP-3	84.7 ± 1.3	20–220 nm (100%)	0.19	−1.37 ± 0.56	50%

## References

[1] Karlson P., Burdette W. B. (1974). Mode of action of ecdysones. *Invertebrate Endocrinology and Hormonal Heterophylly*.

[2] Schmelz E. A., Grebenok R. J., Galbraith D. W., Bowers W. S. (1998). Damage-induced accumulation of phytoecdysteroids in spinach: a rapid root response involving the octadecanoic acid pathway. *Journal of Chemical Ecology*.

[3] Lafont R., Harmatha J., Marion-Poll F., Dinan L., Wilson I. D. (2002). *The Ecdysone Handbook*.

[4] Báthori M., Tóth N., Hunyadi A., Márki Á., Zádor E. (2008). Phytoecdysteroids and anabolic-androgenic steroids. Structure and effects on humans. *Current Medicinal Chemistry*.

[5] Foucault A.-S., Mathé V., Lafont R. (2012). Quinoa extract enriched in 20-hydroxyecdysone protects mice from diet-induced obesity and modulates adipokines expression. *Obesity*.

[6] Oehme I., Bösser S., Zörnig M. (2006). Agonists of an ecdysone-inducible mammalian expression system inhibit Fas Ligand- and TRAIL-induced apoptosis in the human colon carcinoma cell line RKO. *Cell Death and Differentiation*.

[7] Martins A., Tóth N., Ványolós A. (2012). Significant activity of ecdysteroids on the resistance to doxorubicin in mammalian cancer cells expressing the human ABCB1 transporter. *Journal of Medicinal Chemistry*.

[8] Balázs A., Hunyadi A., Csábi J. (2013). ^1^H and ^13^C NMR investigation of 20-hydroxyecdysone dioxolane derivatives, a novel group of MDR modulator agents. *Magnetic Resonance in Chemistry*.

[9] Martins A., Csábi J., Balázs A. (2013). Synthesis and structure-activity relationships of novel ecdysteroid dioxolanes as MDR modulators in cancer. *Molecules*.

[10] Lasic D. D., Barenholz Y. (1996). *Handbook of Nonmedical Applications of Liposomes, Volume 4: From Gene Delivery and Diagnosis to Ecology*.

[11] Ichikawa S., Sugiura S., Nakajima M., Sano Y., Seki M., Furusaki S. (2000). Formation of biocompatible reversed micellar systems using phospholipids. *Biochemical Engineering Journal*.

[12] Kars M. D., Işeri Ö. D., Gündüz U., Ural A. U., Arpaci F., Molnár J. (2006). Development of rational in vitro models for drug resistance in breast cancer and modulation of MDR by selected compounds. *Anticancer Research*.

[13] Pastan I., Gottesman M. M., Ueda K., Lovelace E., Rutherford A. V., Willingham M. C. (1988). A retrovirus carrying an MDR1 cDNA confers multidrug resistance and polarized expression of P-glycoprotein in MDCK cells. *Proceedings of the National Academy of Sciences of the United States of America*.

[14] Chou T. C. (2006). Theoretical basis, experimental design, and computerized simulation of synergism and antagonism in drug combination studies. *Pharmacological Reviews*.

[15] Calcagno A. M., Fostel J. M., To K. K. W. (2008). Single-step doxorubicin-selected cancer cells overexpress the ABCG2 drug transporter through epigenetic changes. *British Journal of Cancer*.

[16] Heffeter P., Pongratz M., Steiner E. (2005). Intrinsic and acquired forms of resistance against the anticancer ruthenium compound KP1019 [indazolium trans-[tetrachlorobis(1H-indazole)ruthenate (III)] (FFC14A). *Journal of Pharmacology and Experimental Therapeutics*.

[17] Heffeter P., Jakupec M. A., Körner W. (2007). Multidrug-resistant cancer cells are preferential targets of the new antineoplastic lanthanum compound KP772 (FFC24). *Biochemical Pharmacology*.

[18] Rotem R., Tzivony Y., Flescher E. (2000). Contrasting effects of aspirin on prostate cancer cells: suppression of proliferation and induction of drug resistance. *The Prostate*.

[19] Konovalova N. P., Mitrokhin Y. I., Volkova L. M., Sidorenko L. I., Todorov I. N. (2002). Ecdysterone modulates antitumor activity of cytostatics and biosynthesis of macromolecules in tumor-bearing mice. *Biology Bulletin of the Russian Academy of Sciences*.

[20] Trubetskoy V. S., Torchilin V. P. (1996). Polyethylene glycol based micelles as carriers of therapeutic and diagnostic agents. *STP Pharma Sciences*.

[21] Cooper G. M. (2000). Lysosomes. *The Cell: A Molecular Approach*.

